# Structure–Activity Relationship of Flavonol O-Methylation Revealed by In Vitro, In Silico and Zebrafish Neurodegeneration Models

**DOI:** 10.3390/ijms27114988

**Published:** 2026-05-30

**Authors:** Kamila Borowiec, Agnieszka Michalak, Katarzyna Targowska-Duda

**Affiliations:** 1 Department of Biotechnology, Microbiology and Human Nutrition, Faculty of Food Science and Biotechnology, University of Life Sciences in Lublin, 20-950 Lublin, Poland; 2 Independent Unit for Behavioral Studies, Faculty of Medical Sciences, Medical University of Lublin, 20-059 Lublin, Poland; 3 Department of Biopharmacy, Faculty of Pharmacy, Medical University of Lublin, 20-059 Lublin, Poland

**Keywords:** isorhamnetin, rhamnetin, antioxidant activity, COX-2 inhibition, molecular docking, oxidative stress, Parkinson’s disease, 6-hydroxydopamine

## Abstract

Flavonols are dietary polyphenols whose biological activity is influenced by structural modifications such as O-methylation. This study compared two quercetin derivatives, isorhamnetin (3′-O-methylquercetin) and rhamnetin (7-O-methylquercetin). Antioxidant activity was evaluated using 2,2-diphenyl-1-picrylhydrazyl (DPPH), 2,2′-azinobis-(3-ethylbenzothiazoline-6-sulphonic acid) (ABTS), cupric reducing antioxidant capacity (CUPRAC), and ferric reducing antioxidant power (FRAP) assays. Cyclooxygenase-2 (COX-2) inhibitory activity was assessed in vitro and supported by molecular docking simulations. In vivo effects included developmental toxicity, behavioral assessment, and locomotor responses in a 6-hydroxydopamine (6-OHDA) model. The results demonstrated that rhamnetin exhibited significantly stronger radical-scavenging and reducing activity in DPPH, ABTS, and FRAP assays, whereas no significant differences were observed in the CUPRAC assay. Isorhamnetin showed stronger COX-2 inhibition, with docking results suggesting a different mode of binding when analyzing possible interactions with enzyme active site. In zebrafish larvae, rhamnetin showed lower observable developmental toxicity within the tested concentration range, whereas isorhamnetin induced developmental abnormalities at higher concentrations. Both flavonols attenuated 6-OHDA-associated locomotor deficits and modulated antioxidant enzyme activity under oxidative stress conditions. In conclusion, our findings indicate that the position of O-methylation influences flavonol antioxidant properties, COX-2 interactions, and organism-level responses.

## 1. Introduction

Chronic diseases, including neurodegenerative disorders such as Alzheimer’s disease (AD) and Parkinson’s disease (PD), represent a major and growing burden in ageing populations. Although their aetiology is multifactorial, progressive neuronal loss is closely associated with oxidative stress and neuroinflammation [1]. Excessive inflammatory responses contribute to neuronal damage via the release of prostaglandins (PG), nitric oxide (NO), and cytokines such as Tumor Necrosis Factor α (TNF-α) and interleukins (IL), driven by dysregulated signalling pathways [2]. Concurrently, overproduction of reactive oxygen species (ROS) and activation of cyclooxygenase-2 (COX-2) further amplify neurodegeneration [1,3].

Flavonoids, particularly flavonols, are widely studied due to their antioxidant and anti-inflammatory properties [4]. They act both as radical scavengers and modulators of endogenous defence systems, while also influencing inflammatory pathways, including COX-2 [5,6]. Isorhamnetin (3,4′,5,7-tetrahydroxy-3′-methoxyflavone) and rhamnetin (3,3′,4′,5-tetrahydroxy-7-methoxyflavone) are naturally occurring methylated derivatives of quercetin present in the human diet [7]. Structural modifications such as O-methylation significantly alter their physicochemical properties and biological activity [8,9]. However, the position-specific effects of O-methylation on flavonol activity remain insufficiently characterized, especially in the context of integrated biological responses.

The zebrafish (*Danio rerio*) model provides a valuable in vivo platform for studying neuroactive compounds, offering conserved neurobiology and high-throughput assessment [10]. In particular, 6-hydroxydopamine (6-OHDA) models reproduce key aspects of dopaminergic neurodegeneration associated with Parkinsonian pathology, although the experimental design should take into account spontaneous regenerative neurogenesis in adult zebrafish [11].

Therefore, this study aimed to compare the impact of positional O-methylation on flavonol activity by evaluating antioxidant, anti-inflammatory, and neuroprotective properties of isorhamnetin and rhamnetin using in vitro assays, molecular docking, and the zebrafish model. This integrative approach was applied to assess the relevance of flavonol methylation patterns for the development of phytopharmaceutical agents targeting neurodegenerative disorders.

## 2. Results

### 2.1. In Vitro Effects of Isorhamnetin and Rhamnetin

#### 2.1.1. Antioxidant and Antiradical Activities

Four complementary assays were employed to evaluate the antioxidant activity of the tested flavonol isomers: 2,2-diphenyl-1-picrylhydrazyl (DPPH) radical scavenging, 2,2′-azinobis-(3-ethylbenzothiazoline-6-sulphonic acid) (ABTS) radical cation scavenging, cupric reducing antioxidant capacity (CUPRAC), and ferric reducing antioxidant power (FRAP). All methods were adapted to a microplate format to enable direct comparison of isorhamnetin and rhamnetin. Antioxidant potency and half-maximal effective concentrations (EC_50_) values are presented in Table 1.

In the DPPH assay, rhamnetin exhibited a significantly lower EC_50_ value (14.88 ± 3.00 µM) than isorhamnetin (31.61 ± 7.14 µM; *p* = 0.0050), indicating higher radical scavenging efficiency. A similar pattern was observed in the ABTS assay, where rhamnetin demonstrated greater antioxidant potency (6.41 ± 1.22 µM vs. 15.26 ± 3.32 µM; *p* = 0.0011). In the FRAP assay, rhamnetin also showed significantly stronger reducing capacity (55.73 ± 11.61 µM vs. 141.61 ± 40.68 µM; *p* = 0.0014). In contrast, no statistically significant difference between the compounds was detected in the CUPRAC assay (*p* = 0.2890), although rhamnetin displayed a numerically lower EC_50_ value (74.60 ± 27.17 µM vs. 90.56 ± 15.43 µM).

Taken together, these results show that positional O-methylation or the resulting limitation of hydroxyl group availability clearly modulates antioxidant activity depending on the redox mechanism involved, highlighting the importance of structural determinants in shaping flavonol reactivity.

#### 2.1.2. COX-2 Inhibition by Isorhamnetin and Rhamnetin

**Figure 1:**
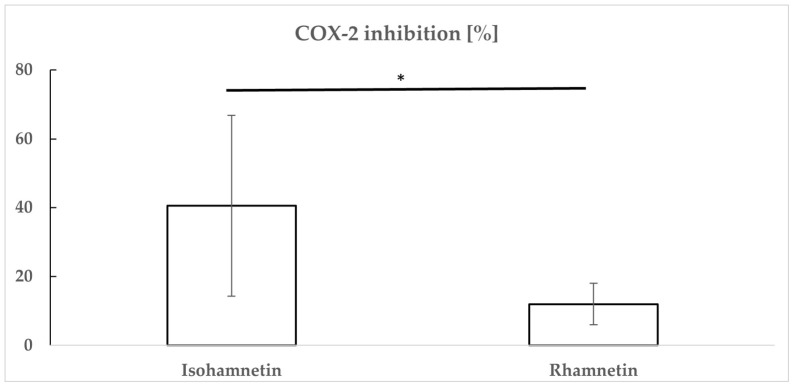
Anti-inflammatory activity of isorhamnetin and rhamnetin expressed as the inhibition of cyclooxygenase-2 (COX-2) at the final concentration of 870 μM. The results presented as mean ± SD (*n* = 8). The *p*-value was calculated using Student’s *t*-test for independent samples (t = 2.62, df = 8, *p* = 0.031), * *p* < 0.05.

Isorhamnetin inhibited COX-2 more strongly (40.54 ± 23.70%) than rhamnetin (11.97 ± 6.00%). The difference was statistically significant (Student’s *t*-test: t = 2.62, df = 8, *p* = 0.031), with a 95% confidence interval for the mean difference of 3.20–53.94% and a large effect size (Cohen’s d = 1.65). Similar significance was observed using the Mann–Whitney test (*p* = 0.032) (Figure 1).

#### 2.1.3. In Silico Analysis of Isorhamnetin and Rhamnetin Interactions with Human COX-2

**Figure 2:**
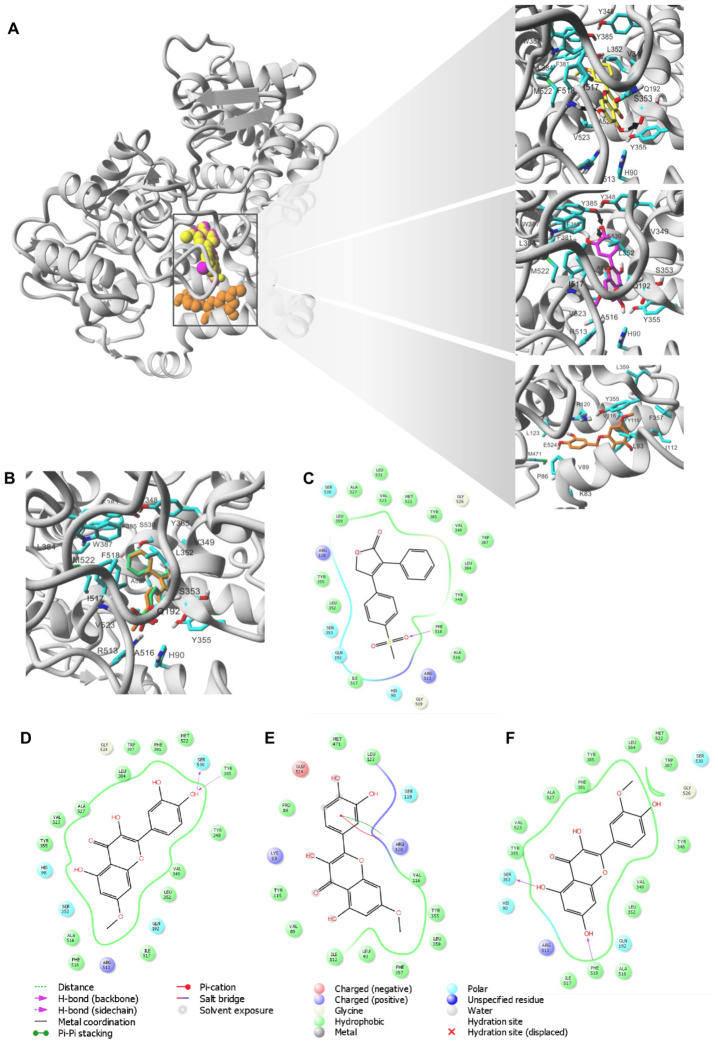
Molecular docking of rhamnetin and isorhamnetin to crystal structure of human COX-2. (**A**) Two orientations of rhamnetin (magenta and orange) and one isorhamnetin orientation (yellow) within the COX-2 binding site; (**B**) 3D map of interactions of rofecoxib (green from pdb crystal structure and orange from docking) with the human COX-2; (**C**) 2D map of interactions of rofecoxib with the human COX-2; (**D**) 2D map of interactions of rhamnetin at lower-energy pos; (**E**) 2D map of interactions of rhamnetin at higher-energy orientation; (**F**) 2D map of interactions of isorhamnetin at COX-2 binding site. 3D representation: hydrogen bonds are marked with black arrows; residues involved in interactions are shown in cyan. Oxygen atoms are colored red, nitrogen blue, phosphorus yellow, and chlorine green. All nonpolar hydrogen atoms are hidden; 2D representation: hydrogen bonds are marked with pink arrows, residues involved in hydrophobic interactions are shown in green, polar in blue, and charged residues involved in binding are shown in red (negative) or purple (positive).

The available crystal structure of human COX-2 was used for docking simulations. To validate the docking protocol, rofecoxib (the ligand from the COX-2 complex) was docked into its experimentally determined binding site. The results confirmed that the ligand occupied the same binding site as shown in the crystal structure (Figure 2B,C). Specifically, the rofecoxib binding site is formed by residues including Ser530, Ser353, Gln192, His90, and Arg513. A hydrogen bond was also suggested between the backbone nitrogen atom (carbonyl moiety) of Phe518 and the oxygen atom of the sulfone group of the ligand. Hydrophobic contacts were observed with Tyr348, Val349, Leu351, Leu352, Tyr355, Leu359, Leu384, Tyr385, Trp387, Ala516, Phe518, Gly519, Met522, and Val523.

Next, rhamnetin and isorhamnetin were docked to the COX-2 structure. Molecular docking results suggested two different orientations of rhamnetin and one orientation of isorhamnetin within the enzyme active site (Figure 2A). An overlapping binding site was observed for both ligands and the reference COX-2 inhibitor, rofecoxib (Figure 2A). The lower-energy site for rhamnetin is formed by residues including His90, Ser353, Gln192, and Arg513. Additionally, one hydrogen bond was suggested between a hydroxyl group of the ligand and the hydroxyl group of Ser530, and a second hydrogen bond between a hydroxyl group of the ligand and the hydroxyl group of Tyr385 (Figure 2A,D). Hydrophobic contacts were observed with Tyr348, Val349, Leu352, Tyr355, Phe381, Leu384, Trp387, Ala516, Ile517, Phe518, Met522, Val523, Gly526, and Ala527.

In the case of the higher-energy pose, the rhamnetin binding site was formed by residues such as Lys83, Ser119, Arg120, and Glu524, while hydrophobic contacts were observed with Pro86, Val89, Leu93, Ile112, Tyr115, Val116, Leu123, Phe357, Leu359, and Met471. Additionally, cation–π interactions were suggested with Arg120 (Figure 2A,E). The predicted binding energy of the lowest-energy rhamnetin complex was –8.6 kcal/mol, whereas the higher-energy complex had a predicted binding energy of −6.0 kcal/mol.

**Figure 3:**
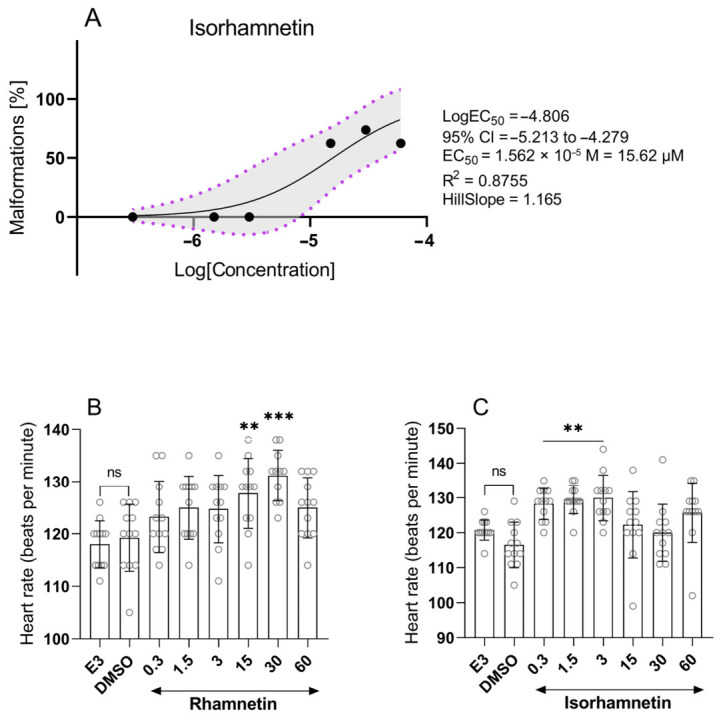
(**A**) EC_50_ malformation for isorhamnetin determined at the final observation time point in the acute toxicity test (4 days post fertilization, dpf). EC_50_ was determined by nonlinear regression analysis, log(agonist) vs. response—variable slope (four parameters). The *x*-axis represents the base-10 logarithm of the concentration expressed in standard units, i.e., M. Purple dotted line—confidence bands, *n* = 24. (**B**) Heart rate of larval zebrafish after 96 h exposure to rhamnetin. (**C**) Heart rate of larval zebrafish after 96 h exposure to isorhamnetin. Heartbeats were counted manually over a period of 10 s and then multiplied by 6 to obtain the result per minute. Data are presented as mean ± SD, Dunn’s multiple comparisons test, ** *p* < 0.01, *** *p* < 0.001, vs. E3, ns, not significant, *n* = 12.

The molecular docking simulations suggested that the isorhamnetin binding site overlapped with the lower-energy site proposed for rhamnetin (Figure 3A,F). Two hydrogen bonds were also suggested, but with different residues than those observed for rhamnetin. Specifically, one hydrogen bond was formed between the hydroxyl group of isorhamnetin and the hydroxyl group of Ser353, and the second between the backbone nitrogen atom (carbonyl moiety) of Phe518 and a hydroxyl group of the ligand. The predicted binding energy of the isorhamnetin complex was −9.6 kcal/mol, while that of the rofecoxib complex was −10.3 kcal/mol. Rofecoxib exhibited substantially more favorable docking scores, consistent with its role as a reference COX-2 inhibitor. It should also be noted that docking scores provide relative estimates of binding favorability and are not directly proportional to experimental inhibitory potency.

### 2.2. Biological Activity of Isorhamnetin and Rhamnetin in Embryonic–Larval Zebrafish

#### 2.2.1. Toxicological Profile

**Figure 4:**
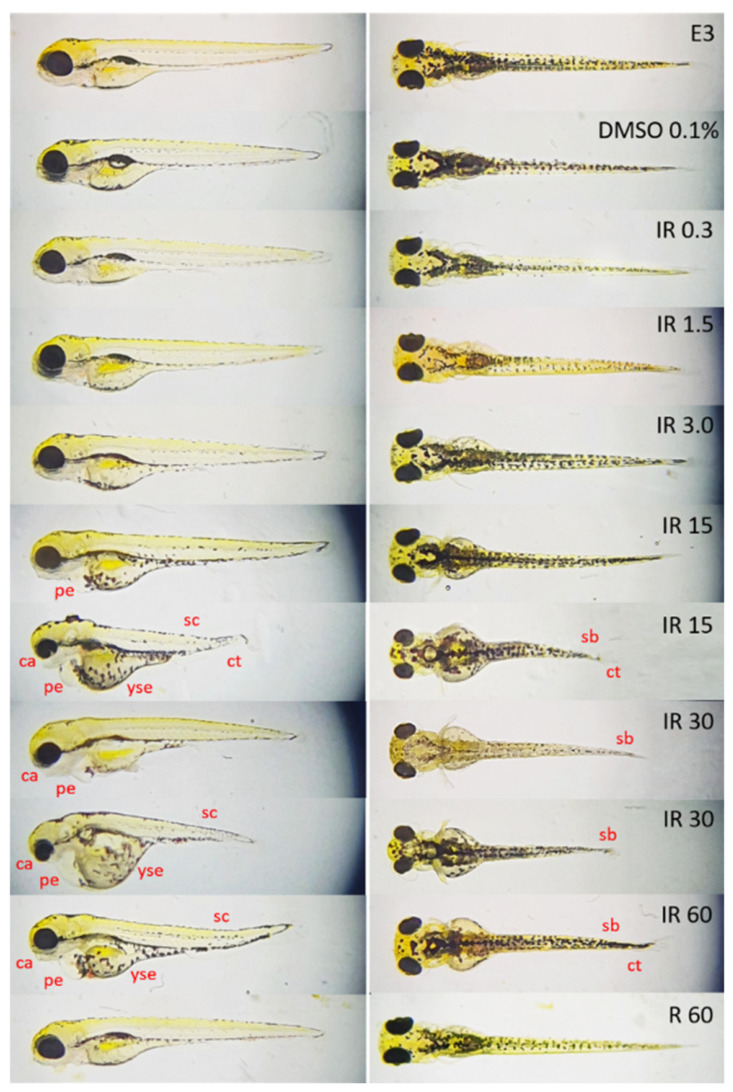
Zebrafish malformations determined at the final observation time point in the acute toxicity test (4 dpf). Panel includes negative control—E3, solvent control—DMSO 0.025%, all tested concentrations of isorhamnetin (IR, 0.3–60 µM) as well as the highest concentration of rhamnetin (R, 60 µM); ca: craniofacial anomalies, ct, curved tail, pe, pericardial edema, sb, shortened body, sc, spinal curvature, yse, yolk sac edema.

Representative effects of rhamnetin and isorhamnetin on larval zebrafish are shown in Figure 3. Due to the limited stability of both compounds in low-concentration aqueous dimethyl sulfoxide (DMSO) solutions and solvent constraints, the median lethal concentration (LC_50_) could not be reliably determined under the experimental conditions. Rhamnetin, at concentrations ranging from 0.3 to 60.0 µM, did not induce observable malformations, or their incidence was below 10%, which is considered acceptable for the negative control group. In contrast, isorhamnetin caused pronounced body malformations in larval zebrafish starting at 15 µM, with the EC_50_ for malformation calculated as 15.62 µM (Figure 3A). Neither flavonol affected the hatching rate; however, both compounds elicited a significant increase in heart rate, although at different concentration ranges (Figure 3B,C). Representative examples of the most frequently observed malformations are shown in Figure 4.

#### 2.2.2. Locomotor Activity and Anxiety-Like Behaviours

**Figure 5:**
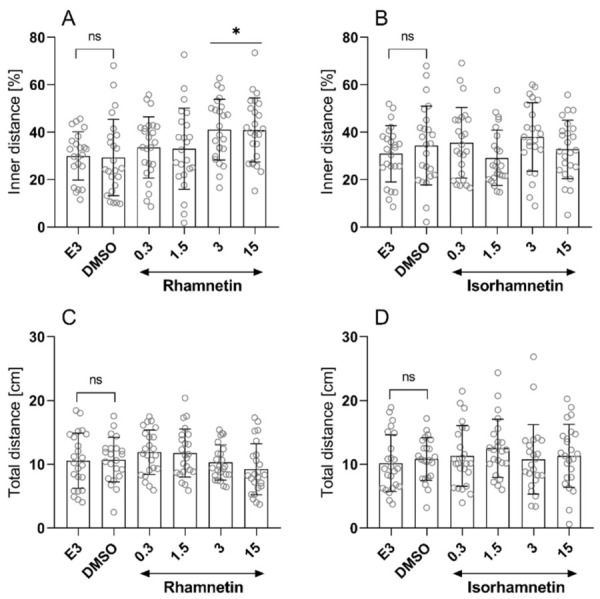
(**A**,**C**) Anxiety- and locomotor-related response in 6 dpf zebrafish larvae after 24 h exposure to rhamnetin. (**B**,**D**) Anxiety- and locomotor-related response in 6 dpf zebrafish larvae after 24 h exposure to isorhamnetin. Zebrafish behaviours were assessed within first minute after light transition. Data are presented as mean ± SD, Dunnett’s multiple comparisons test, * *p* < 0.05, vs. E3, ns, not significant, *n* = 24.

The effects of rhamnetin and isorhamnetin on locomotor activity and anxiety-like behaviors were assessed in 6-day post-fertilization (dpf) zebrafish larvae following 24 h exposure. Neither rhamnetin nor isorhamnetin affected locomotor activity at the tested concentrations (0.3–15.0 µM) (Figure 5C,D). Rhamnetin, however, exhibited anxiolytic-like effects, as indicated by an increase in the distance traveled in the inner zone, reflecting a reduction in thigmotactic behavior, a common measure of anxiety-like responses (Figure 5A,B).

#### 2.2.3. Neuroprotection Against 6-OHDA Toxicity

**Figure 6:**
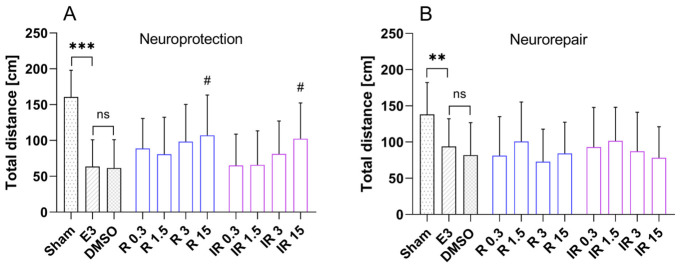
(**A**) Locomotor activity after 48 h exposure to 6-hydroxydopamine (6-OHDA) completed with exposition to tested flavonols for 48 h in the neuroprotection assay. (**B**) Locomotor activity after 48 h exposure to 6-OHDA completed with exposition to tested flavonols for 24 h in the neurorepair assay. Locomotor activity, expressed as the total distance travelled, was measured during the 10-min dark period. Data are presented as mean ± SD. Tukey multiple comparisons test, ** *p* < 0.01, *** *p* < 0.001, vs. Sham, Dunnett’s multiple comparisons test # *p* < 0.05, vs. E3, ns, not significant, *n* = 24; R, the group co-treated with 6-OHDA and rhamnetin (0.3–15 µM); IR, the group co-treated with 6-OHDA and isorhamnetin (0.3–15 µM); sham, the control group not treated with 6-OHDA; E3, the control group treated with 6-OHDA; DMSO, the group co-treated with 6-OHDA and DMSO.

**Figure 7:**
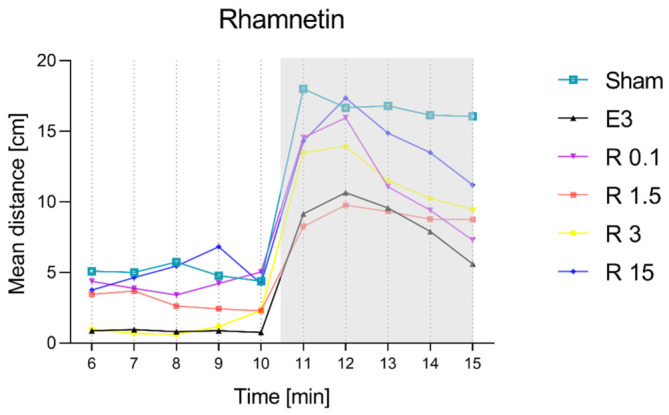
Locomotor responses to light/dark transitions in zebrafish larvae exposed to the 6-OHDA in neuroprotection assay, treated with rhamnetin (R; (0.3–15 µM). Figure represents the last 5 min of the light period and the first 5 min of the dark period. Data are presented as mean ± SD, *n* = 24.

The effects of isorhamnetin and rhamnetin on locomotor deficits associated with 6-OHDA exposure were evaluated. Both flavonols, at the highest tested concentration (15 µM), attenuated locomotor deficits associated with 6-OHDA exposure, as reflected by improved behavioral performance (Figure 6A), although no neurorestorative potential was observed (Figure 6B). Moreover, rhamnetin at 15 µM (R15) elicited a locomotor response to the light–dark transition comparable to that of the control sham group (not treated with 6-OHDA), significantly reversing the 6-OHDA-induced flattening of the curve slope after light transition observed in the E3 group (Figure 7). A significant increase in the distance traveled by the larvae, compared with the E3 group, was observed at minutes 11, 12, 14, and 15 of the assay, corresponding to 1, 2, 4, and 5 min of the dark phase following the light-to-dark transition (*p* < 0.05).

### 2.3. Oxidative Stress and Antioxidant Enzyme Evaluation in Zebrafish Larvae

Larvae used to assess the neuroprotective potential of flavonols were used for further analyses. Homogenates were prepared from pooled larvae, and protein concentrations were determined using the Pierce™ Bicinchoninic Acid (BCA) assay to normalize enzymatic activities per mg of protein. The mean protein concentration was 0.74 ± 0.06 mg/mL of homogenate.

**Figure 8:**
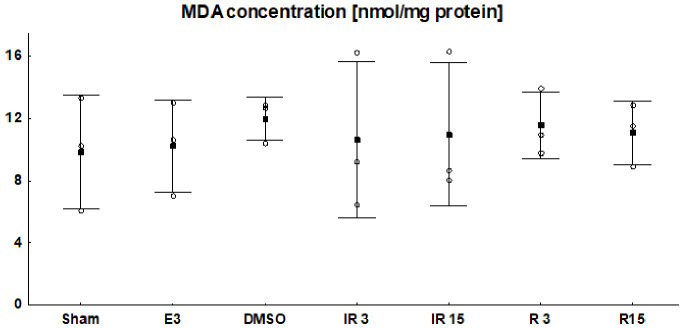
Individual data points and box plots of malondialdehyde (MDA) concentration in homogenates prepared from 6 dpf zebrafish larvae (*n* = 20) in the control group (sham) and in the tested groups after exposure to 6-OHDA in the neuroprotection assay (E3 or DMSO) and co-treatment with isorhamnetin (IR3 and IR15) or rhamnetin (R3 and R15). Data are presented as mean ± SD (*n* = 3). Squares represent the mean, and whiskers indicate SD. Raw data are shown as circles.

Malondialdehyde (MDA) levels, assessed as a marker of lipid peroxidation, were comparable across all experimental groups, including sham, 6-OHDA-treated larvae, and those co-treated with isorhamnetin or rhamnetin (Figure 8). Box plot analysis showed substantial overlap between groups, and the Kruskal–Wallis test confirmed no significant differences (H(6,21) = 1.13, *p* = 0.98; *n* = 3), indicating that neither 6-OHDA exposure nor flavonol treatment significantly affected lipid peroxidation.

**Figure 9:**
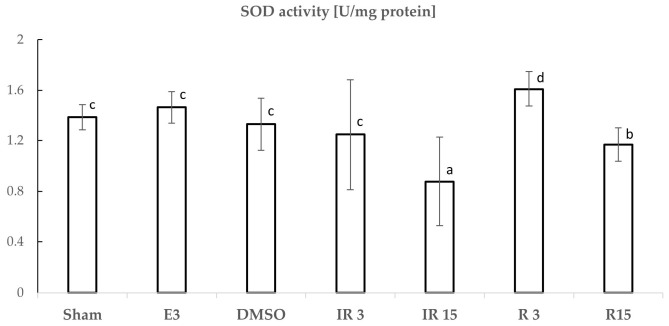
Superoxide dismutase (SOD) activity in homogenates prepared from 6 dpf zebrafish larvae (*n* = 20) in the control group (sham) and in the tested groups after exposure to 6-OHDA in the neuroprotection assay (E3 or DMSO) and co-treatment with isorhamnetin (IR3 and IR 5) or rhamnetin (R3 and R15). Data are presented as mean ± SD (*n* = 5; Tukey’s HSD test; homogenous groups, alpha = 0.05) The results sharing a common letter do not differ significantly at *p* < 0.05.

In contrast, significant differences were observed in antioxidant enzyme activities. Superoxide dismutase (SOD) activity (U/mg protein; *n* = 5) differed significantly among groups (one-way analysis of variance, ANOVA, *p* < 0.05; Figure 9). The lowest activity was recorded in the group co-treatment with 15 µM isorhamnetin (0.88 U/mg), which was significantly lower than in all other groups. The group co-treatment with 15 µM rhamnetin showed intermediate activity (1.17 U/mg). The control sham group as well as the 6-OHDA groups co-treated with DMSO, E3 and 3 µM isorhamnetin exhibited comparable SOD activities (1.33–1.46 U/mg). The highest activity was observed in the group co-treated with 3 µM rhamnetin (1.61 U/mg).

**Figure 10:**
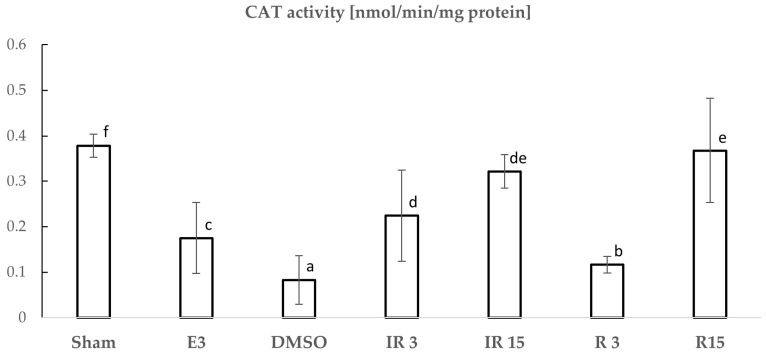
Catalase (CAT) activity in homogenates prepared from 6 dpf zebrafish larvae (*n* = 20) in the control group (sham) and in the tested groups after exposure to 6-OHDA in the neuroprotection assay (E3 or DMSO) and co-treatment with isorhamnetin (IR3 and IR15) or rhamnetin (R3 and R15). Data are presented as mean ± SD (*n* = 4; Tukey’s HSD test; homogenous groups, alpha = 0.05) The results sharing a common letter do not differ significantly at *p* < 0.05.

Catalase (CAT) activity (U/mg protein; *n* = 4) also differed significantly among groups (ANOVA, *p* < 0.05; Figure 10) and followed a pattern distinct from that observed for SOD. The lowest CAT activity was detected in the groups co-treated with DMSO (0.083 ± 0.053 U/mg) and 3 µM rhamnetin (0.117 ± 0.018 U/mg), with no significant difference between them. Intermediate activities were observed in the groups co-treated with E3 (0.175 ± 0.078 U/mg) and 3 µM isorhamnetin (0.224 ± 0.101 U/mg), followed by the highest tested concentrations of isorhamnetin and rhamnetin (0.322 ± 0.037 U/mg and 0.368 ± 0.115 U/mg, respectively), which did not differ significantly from each other. The highest CAT activity was recorded in the sham group (0.378 ± 0.025 U/mg), significantly higher than in all other experimental groups.

Overall, these results indicate that flavonol treatment modulates antioxidant enzyme activities under conditions of 6-OHDA-induced oxidative stress in an enzyme- and dose-dependent but nonlinear manner, whereas lipid peroxidation remains unchanged. Namely, low-dose rhamnetin markedly enhanced SOD activity, whereas high-dose rhamnetin strongly restored CAT activity to near-control levels. Isorhamnetin showed a weaker but dose-dependent increase in CAT activity. These results suggest a dose-dependent shift in antioxidant defense from superoxide scavenging toward hydrogen peroxide detoxification as well as stronger and broader antioxidant potential of rhamnetin.

## 3. Discussion

A set of complementary antioxidant assays (DPPH, ABTS, FRAP, CUPRAC) was applied to compare redox properties of structurally related flavonols across different mechanisms, including hydrogen atom transfer (HAT) and single-electron transfer (SET). While FRAP and CUPRAC assess reducing capacity under distinct conditions, DPPH and ABTS provide insight into radical-scavenging activity [12]. As these assays represent simplified chemical systems, they were used here for mechanistic comparison of two isomers.

Within this framework, positional O-methylation clearly influenced antioxidant activity in a mechanism-dependent manner. Rhamnetin exhibited consistently higher activity than isorhamnetin in DPPH, ABTS, and FRAP assays, whereas no difference was observed in the CUPRAC assay. This pattern reflects the importance of the B-ring catechol system (C3′, C4′) for electron delocalization [13]. Methylation at C3′ disrupts this system and reduces radical-scavenging efficiency [14], whereas methylation at C7 preserves it [15]. The lack of differences in the CUPRAC assay suggests that the impact of methylation depends on the redox environment, as this assay is an electron-transfer-based method involving the reduction of the Cu(II)–neocuproine complex at near-physiological pH [16], which emphasizes overall reducing capacity rather than specific structural features such as catechol moiety.

Given the relevance of neuroinflammation, COX-2 was selected as a molecular target. Docking simulations indicated that both flavonols bind within the canonical non-steroidal anti-inflammatory drug (NSAID)-binding channel, interacting with key residues (e.g., Ser530, Ser353, His90, Arg513, Tyr385, Trp387, Phe518). However, docking results should be interpreted cautiously as supportive rather than definitive, particularly for polyphenols capable of nonspecific interactions. The predicted differences in binding energies between isorhamnetin and rhamnetin were relatively small; such differences should be interpreted with caution and are not necessarily directly proportional to biological potency. In this context, the docking results should be considered supportive of the experimental findings rather than predictive of quantitative differences in inhibitory potency. Therefore, the docking analysis was interpreted mainly at the level of binding orientation and interaction patterns rather than absolute thermodynamic ranking. In this context, the slightly more favorable predicted binding of isorhamnetin, together with its interaction profile within the canonical COX-2 channel, remained broadly consistent with the experimentally observed higher COX-2 inhibitory activity relative to rhamnetin. Moreover, ligands were treated as flexible during docking, but their conformational space is limited by the largely planar flavonol scaffold. The receptor was also treated as rigid, which does not account for active-site flexibility or induced-fit effects.

Consistent with in silico predictions, isorhamnetin showed stronger COX-2 inhibition than rhamnetin, although only at relatively high concentrations, supporting a comparative interpretation and reflecting differences between the tested flavonols. After all, physiologically achievable plasma levels in vivo following oral flavonol intake are typically reported in the low micromolar range [17]. This discrepancy is largely associated with the poor aqueous solubility and limited bioavailability characteristic of flavonols [18,19]. Therefore, the observed effects should be interpreted primarily in a comparative and mechanistic context rather than as evidence of direct pharmacological potency. Moreover, at higher concentrations, nonspecific interactions with membranes, proteins, and transporters may contribute to the observed effects [20].

Together, these findings suggest that positional O-methylation at C3′ reduces redox activity and may contribute to differences in ligand orientation and interaction patterns within the COX-2 catalytic channel. This aligns with the literature suggesting that isorhamnetin primarily affects inflammatory signaling pathways and COX-2 expression [21], while rhamnetin also exhibits anti-inflammatory potential [22]. Structural considerations further support this interpretation, as COX-2 binding is influenced by hydroxyl groups (C4′, C7) and hydrophobic substituents such as C3′-OCH_3_ [23], supporting a greater contribution of isorhamnetin (C7-OH; C4′-OH; C3′-OCH_3_) relative to rhamnetin (only C4′-OH).

Importantly, the differential COX-2 binding behavior of rhamnetin and isorhamnetin does not appear to arise from the total number of hydrophobic or hydrogen-bond interactions but rather from their distinct binding orientations within the NSAID channel. In particular, docking simulations suggested that isorhamnetin more frequently adopted poses preserving simultaneous interactions with key catalytic residues (Ser353, Ser530, Tyr385, and Phe518), whereas rhamnetin shows greater variability in docking poses. Thus, the most decisive structural criterion explaining the observed selectivity seems to be the ability to maintain interactions with key catalytic residues within the canonical COX-2 catalytic channel geometry rather than the absolute number of contacts. In addition, C3′-O-methylation modulates the spatial distribution of hydrogen-bond donors/acceptors and enhances local hydrophobic complementarity, which may further stabilize the preferred binding orientation of isorhamnetin. In addition to electronic effects associated with disruption of the catechol system, O-methylation alters key physicochemical parameters. Methoxylation generally increases lipophilicity (calculated logarithm of the octanol/water partition coefficient, cLogP) and reduces hydrogen-bond donor capacity, potentially enhancing passive membrane permeability while maintaining relatively high polarity due to preserved hydroxyl groups [24]. Consistently, physicochemical analysis (SwissADME) showed that isorhamnetin and rhamnetin possess identical global properties, including consensus cLogP (~1.1) and topological polar surface area (TPSA; 120.36 Å^2^), indicating comparable lipophilicity, polarity, and predicted membrane permeability [25] (Figure S1). Despite these similarities, the two isomers differ in hydroxyl group positioning, which influences their pharmacochemical profiles. These observations suggest that the biological differences may be more strongly associated with local electronic effects and substituent positioning than with global physicochemical parameters. In particular, O-methylation at the C3′ position in isorhamnetin likely enhances hydrophobic interactions within the COX-2 binding channel, consistent with its higher inhibitory activity, whereas rhamnetin preserves the catechol arrangement associated with stronger electron-donating and antioxidant properties [26].

To evaluate whether these molecular differences translate into organism-level effects, a zebrafish larval model was used. The interpretation of comparative toxicity profiles should be approached cautiously because LC_50_ values could not be reliably determined under the applied experimental conditions due to limited aqueous solubility of flavonols [19,27]. At higher nominal concentrations, incomplete dissolution or partial precipitation may have affected actual exposure concentrations in zebrafish media [28]. Therefore, the observed differences between rhamnetin and isorhamnetin should be interpreted primarily as differences in observable developmental responses within the tested concentration range rather than definitive differences in safety margins.

Nevertheless, rhamnetin showed no developmental toxicity within 0.3–60 µM, whereas isorhamnetin induced malformations from 15 µM (EC_50_ = 15.62 µM), indicating higher developmental toxicity under the applied experimental conditions. Neither flavonol affected the hatching rate; however, both compounds increased heart rate at different concentration ranges. The increase in heart rate is interpreted as a non-specific physiological response, which may reflect mild pharmacological stress or off-target effects rather than a clearly adaptive mechanism, as heart rate is a sensitive but non-specific endpoint in zebrafish-based cardiovascular screening [29]. Rhamnetin exhibited anxiolytic-like effects, as reflected by reduced thigmotactic behaviour in zebrafish [30], and the absence of changes in baseline locomotor activity argues against general motor impairment as a confounding factor. Together, these findings support an anxiolytic-like effect under the applied experimental conditions. However, this observation should be interpreted with caution and considered as a preliminary indication of anxiolytic activity. Next, both flavonols attenuated 6-OHDA-induced locomotor deficits under oxidative stress conditions. Notably, rhamnetin more effectively restored the light–dark response, approaching control levels. No neurorestorative effects were observed, indicating functional neuroprotection (i.e., preservation of neuronal function) rather than structural neuronal recovery or regeneration. Considering that 6-OHDA induces mitochondrial dysfunction and ROS overproduction [31], the observed effects may be associated with modulation of redox homeostasis and potentially related signalling pathways. However, given that the 6-OHDA zebrafish model reflects a complex and multi-mechanistic pathological process, the present data do not allow discrimination between direct ROS-scavenging effects and modulation of neuroinflammatory pathways as different classes of compounds acting through distinct mechanisms can produce similar functional rescue in this model [11]. Taken together, these findings provide functional behavioral and biochemical evidence consistent with neuroprotective-related activity at the organism level; however, direct neuronal preservation was not assessed in the present study and warrants further investigation in future mechanistic studies.

Importantly, despite its slightly stronger predicted interaction with COX-2, isorhamnetin exhibited a less favorable developmental safety profile in zebrafish. Such discrepancies are not unexpected, as docking reflects affinity toward a single molecular target, whereas organism-level responses result from broader biological interactions. In addition to inflammatory signaling [32], isorhamnetin has been reported to modulate multiple cellular pathways, oxidative stress, apoptosis, and cell cycle regulation [33], indicating pleiotropic biological activity. Therefore, the higher developmental toxicity observed for isorhamnetin may reflect combined perturbation of multiple regulatory networks rather than COX-2-related effects alone. In particular, methylation at the C3′ position in isorhamnetin alters the spatial distribution of hydroxyl groups and disrupts the catechol structure, which may also affect redox balance and interactions with multiple cellular targets during early development, potentially contributing to the higher incidence of developmental abnormalities.

Further insight into these multi-level effects is provided by the biochemical analyses. Although MDA levels remained unchanged, significant modulation of antioxidant enzyme activities was observed. Changes in SOD and CAT activities indicate adjustment of redox homeostasis. Notably, rhamnetin produced a stronger overall antioxidant response, with low doses markedly increasing SOD activity and higher doses restoring CAT activity toward control levels, whereas isorhamnetin induced a weaker but dose-dependent increase in CAT activity. Together, these findings indicate that positional O-methylation determines distinct and complementary antioxidant response profiles. This is consistent with coordinated antioxidant defense mechanisms, where SOD and CAT act sequentially as a primary enzymatic barrier to limit ROS accumulation and prevent downstream oxidative damage [34,35]. The lack of MDA changes further suggests that single markers of lipid peroxidation may not fully reflect oxidative stress dynamics. However, this observation should be interpreted with caution given the limited sample size, which may reduce the sensitivity to detect subtle alterations in lipid peroxidation, particularly in the context of inherently variable biomarkers such as MDA and its methodological limitations [36]. Additionally, the observed nonlinear patterns may reflect a hormetic-like response, which is commonly observed in antioxidant systems under oxidative stress conditions [37].

Our findings align with previous zebrafish studies showing that flavonoids such as quercetin primarily stabilize redox and inflammatory balance rather than induce dopaminergic regeneration [38]. Together with evidence of tissue-specific antioxidant responses [39], this highlights the importance of systemic redox regulation in shaping neuroprotective outcomes. Importantly, the larval zebrafish model does not fully recapitulate the complexity of mammalian dopaminergic systems, particularly with regard to mature nigrostriatal organization, regulatory feedback loops [40], and the difference in the regenerative capacity [41]. Therefore, extrapolation of these findings to mammalian neurodegeneration should be made with caution.

## 4. Materials and Methods

### 4.1. Standards

Isorhamnetin and rhamnetin were purchased from Merck (Darmstadt, Germany). Stock solutions (6 mM in 100% DMSO) were prepared and diluted in appropriate solvents, including ethanol, methanol, or water, depending on the analytical requirements of each assay. Final concentrations used in the experiments are specified in the descriptions of the individual methods.

### 4.2. In Vitro Antioxidant Capacity of Isorhamnetin and Rhamnetin Using ABTS, DPPH, CUPRAC, and FRAP Assays

Antioxidant activity of rhamnetin and isorhamnetin standards (stock solutions in DMSO); final concentrations 0.5–1000 µM in methanol or ethanol) was determined using the DPPH [42], ABTS [43], CUPRAC [44], and FRAP [45] assays according to previous reports with minor modifications. Concentration–response curves were constructed, and EC_50_ were calculated as the concentration producing 50% of the maximal response; details of regression analysis are provided in the Statistics (Section 4.6).

For the DPPH assay, 50 μL of standard (in methanol, *n* = 4) was mixed with 70 μL methanol and 180 μL DPPH (2 mM, methanol). After shaking and incubation in the dark, absorbance (A_1_) was measured at 515 nm at 0 min and every 15 min until a plateau was reached (120 min) at 21 °C using a Varioskan™ LUX multimode microplate reader (Thermo Fisher Scientific, Waltham, MA, USA). Control samples (A_2_) contained DMSO:methanol. Radical scavenging capacity was expressed as percentage effect (E% = (A_2_ − A_1_)/A_2_ × 100%).

For the ABTS assay, 100 μL of standard (in ethanol, *n* = 4) was combined with 200 μL ABTS solution (7 mM containing 2.45 mM potassium persulfate; incubated for 24 h in the dark and diluted 20-fold to obtain an absorbance of approximately 1.2 at 734 nm). Absorbance (A_1_) was recorded at 734 nm for 30 min at 2 min intervals (21 °C, Varioskan™ LUX multimode microplate reader). Control samples (A_2_) contained DMSO:ethanol, and results were expressed as percentage effect (E%).

In the CUPRAC assay, 40 μL of standard (in ethanol, *n* = 5) was mixed with 60 μL ammonium acetate buffer (1 M, pH 7.0), 50 μL neocuproine (7.5 mM, water:acetonitrile 1:1), and 50 μL copper (II) chloride (10 mM). After 1 h, absorbance (A_1_) was measured at 450 nm (21 °C, Varioskan™ LUX multimode microplate reader). Control samples (A_2_; DMSO:ethanol) were included in the calculations (A_1_ − A_2_).

For the FRAP assay, 10 μL of standard (in ethanol, *n* = 5) was added to 190 μL FRAP reagent composed of acetate buffer (300 mM, pH 3.6), 2,4,6-tripyridyl-S-triazine (TPTZ, 40 mM) in hydrochloric acid (40 mM), and iron (III) chloride hexahydrate (40 mM). The reagent was pre-warmed at 37 °C for 10 min prior to use. After 30 min incubation at 37 °C in the dark, absorbance was recorded at 593 nm (Varioskan™ LUX multimode microplate reader). Control samples (A_2_) contained DMSO:ethanol.

### 4.3. Combined Experimental and In Silico Evaluation of COX-2 Inhibitory Effects of Rhamnetin and Isorhamnetin

#### 4.3.1. COX-2 Inhibitory Activity Screening Assay

The inhibition of human recombinant COX-2 by the test compounds was evaluated using a COX inhibitor screening assay kit (Cayman Chemical, Ann Arbor, MI, USA). Stock solutions of the standards were diluted in ethanol, and the final concentrations of the tested standards in the assay mixture were 435–870 μM, respectively (*n* = 5), and the assay was performed according to the manufacturer’s instructions (see Table S1 for details).

#### 4.3.2. Molecular Docking to Crystal Structure of Human COX-2

The available crystal structure of human COX-2 in complex with rofecoxib at 2.70 Å resolution (PDB ID: 5KIR) was used for molecular docking simulations [46]. Rofecoxib was one of the first COX-2 inhibitors. Rhamnetin and isorhamnetin were retrieved from the PubChem database in Molfile format and optimized using the semi-empirical AM1 method implemented in Spartan 10 (Wavefunction, Inc., Irvine, CA, USA), as previously described [47,48,49]. The optimized structures were subsequently used for ligand docking. Docking simulations of the flexible ligands into the substrate/inhibitor pocket of the COX-2 crystal structure were performed using AutoDock Vina v1.2.0 (The Scripps Research Institute, La Jolla, CA, USA) [50]. The grid box was generated using MGLTools 1.5.6 (The Scripps Research Institute, La Jolla, CA, USA). The grid dimensions were set to 18 Å × 18 Å × 18 Å, with a grid-point spacing of 1 Å to cover the cyclooxygenase channel. AutoDock Vina parameters were set as follows: exhaustiveness = 100 and number of modes = 20. To verify the docking protocol, rofecoxib was docked into COX-2 active site to compare and validate poses with its experimentally determined binding mode. The lowest-energy conformations were selected from clusters of superposed poses generated for each ligand docked into the COX-2 binding site.

### 4.4. In Vivo Study of Zebrafish Model

#### 4.4.1. Animals

Zebrafish (AB strain) larvae were maintained and used according to protocols previously described by Kurach and colleagues [10], specifically, embryos were cultured under standard laboratory conditions: temperature 28 ± 0.5 °C, a 14/10 h light/dark cycle, and E3 medium. All experimental procedures were carried out at the Experimental Medicine Centre of the Medical University of Lublin (Poland), in accordance with both EU and national regulations concerning the care and use of laboratory animals.

**Figure 11:**
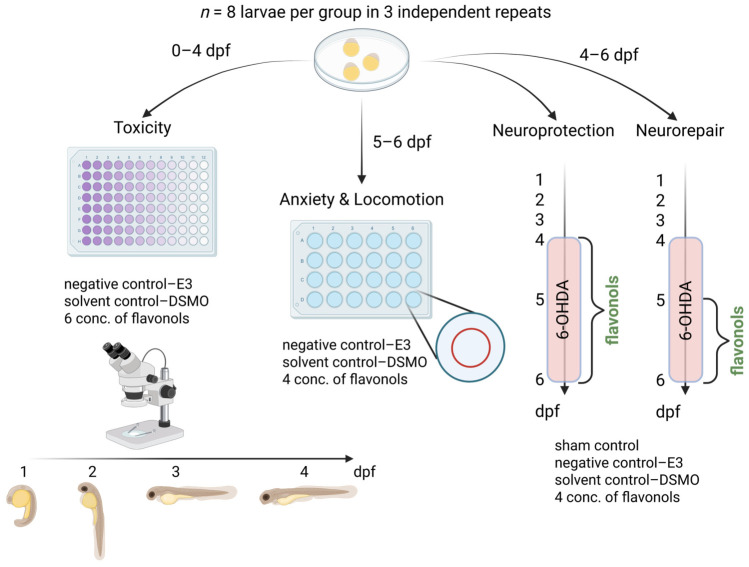
General experimental scheme of in vivo studies using the zebrafish embryo–larvae model for the evaluation of toxicity, anxiety-like behaviors, general locomotor activity, and neuroprotective and neurorepair effects of 6-OHDA. Arrows indicate different experimental paradigms, while, numbers (1–6) represent days post-fertilization (dpf). Created in BioRender. Michalak, A. (BioRender Inc., Toronto, ON, Canada; https://BioRender.com/frt43z7) is licensed under CC BY 4.0; 29 May 2026).

For the toxicity study, 336 zebrafish embryos/larvae were used from 0 to 4 days post fertilization (dpf) (*n* = 8 larvae per group in 3 independent repeats), 288 larvae were subjected to testing of anxiety and locomotor activity (*n* = 8 larvae per group in 3 independent repeats), and 576 larvae were subjected to testing of neuroprotection and neurorepair (*n* = 8 larvae per group in 3 independent repeats). Studies using zebrafish larvae older than 5 dpf were performed with the approval of the Local Ethics Committee for Animal Experimentation in Lublin (Lokalna Komisja Etyczna do Spraw Doświadczeń na Zwierzętach w Lublinie), University of Life Sciences in Lublin, Poland (approval no. 96/2022). Following the completion of all analyses, larvae were humanely euthanized using tricaine (300–500 mg/L). The general scheme of in vivo zebrafish testing is presented in Figure 11.

#### 4.4.2. Analysis of Toxicity in Zebrafish Embryos/Larvae

The zebrafish embryo acute toxicity test was performed according to OECD Test Guideline 236 [51]. Briefly, fertilized eggs were exposed to the test compounds for 96 h. Lethality was assessed every 24 h based on four indicators: egg coagulation, absence of somite formation, failure of tail–bud detachment, and lack of heartbeat. Acute toxicity was determined by the presence of any of these effects. General toxicity was further evaluated based on the occurrence of malformations. The EC_50_ was calculated from the incidence of embryonic malformations [10,51]. Solutions of isorhamnetin and rhamnetin were tested at six concentrations (0.3–60.0 µM) with a final DMSO concentration of 0.025%. Due to limited aqueous solubility of flavonols, complete dissolution at higher concentrations could not be guaranteed throughout the exposure period. Importantly, the tested concentrations were limited to the range in which solutions remained visually clear, indicating adequate compound dissolution under the applied experimental conditions.

#### 4.4.3. Locomotor and Anxiety-Like Behaviours in Zebrafish Larvae

The methodology was developed at the Independent Behavioural Research Laboratory of the Medical University of Lublin and is based on the previous protocol [52]. Specifically, 5 dpf larvae were individually transferred to 24-well plates. After 24-h incubation in the test solution (0.3–15 µM; 0.025% DMSO), larvae were subjected to behavioral analysis using the DanioVision system (Noldus Information Technology, Wageningen, The Netherlands) with EthoVision XT (Noldus Information Technology, Wageningen, The Netherlands) for video tracking. The larvae were exposed to continuous light for 6 min, followed by a 4-min dark phase. Anxiety-like behavior was assessed based on thigmotaxis, expressed as the percentage of distance travelled within the central area of the well during the first minute after the light transition. Total distance travelled was simultaneously measured to evaluate locomotor activity.

#### 4.4.4. Analysis of Neuroprotection and Neurorepair in the 6-OHDA-Induced Neurotoxicity in Zebrafish Larvae

The procedure was adapted from a previously published protocol [11]. Specifically, 4 dpf larvae were exposed for 48 h to 6-OHDA at a concentration of 250 µM, prepared in a 0.01% ascorbic acid solution. This procedure was not performed on the sham control group, which served as a negative control to confirm the neurodegenerative effect of 6-OHDA. The procedure was carried out on all remaining experimental groups. Simultaneously, the larvae were treated with the tested flavonol solutions at four concentrations (0.3–15 µM; 0.025% DMSO) in three independent replicates to evaluate their neuroprotective (from 4 dpf) or neurorepair (from 5 dpf) potential.

After the incubation period, larvae were transferred to 96-well plates and placed in the apparatus used for behavioral testing for a 30-min acclimation period. They were exposed to continuous light for 10 min, followed by a 10-min dark phase. Neurodegeneration was assessed based on changes in locomotor activity, measured as the total distance travelled during the dark phase.

### 4.5. In Vitro Biochemical Assays for Oxidative Stress and Antioxidant Capacity in Zebrafish Larvae Homogenates

#### 4.5.1. Tissue Homogenization

Approximately 20 zebrafish larvae (~20 mg tissue, as described in Section 4.4.4.) originating from the same treatment groups were collected and pooled to obtain sufficient biological material for biochemical analyses. The larvae were derived from three independent biological experiments (*n* = 8 larvae per group per experiment). Tissue samples were placed in 2.0 mL prefilled tubes containing 0.5 mm glass beads suitable for tissue homogenization (Canvax Reagents SL, Valladolid, Spain) and homogenized on ice in 600 µL of cold MDA lysis buffer (Lipid Peroxidation (MDA) Assay Kit, Sigma-Aldrich, St. Louis, MO, USA) containing 6 µL of butylated hydroxytoluene (BHT). Tissue disruption was performed using a TissueLyser instrument (Qiagen, Hilden, Germany) at 25–30 Hz for two cycles of 1 min each, with a 30–60 s cooling interval on ice between cycles to prevent temperature-induced degradation of analytes. Following homogenization, samples were visually inspected to confirm complete tissue lysis and subsequently centrifuged at 13,000× *g* for 10 min at 4 °C. The resulting supernatants were aliquoted into multiple low-binding microtubes (20–50 µL per aliquot) and immediately stored at −80 °C until analysis.

#### 4.5.2. BCA, MDA, SOD and CAT Assays

Biochemical analyses were performed using homogenates prepared from pooled zebrafish larvae, as described above. The assays were conducted in technical replicates according to the respective assay protocols. Protein concentrations were determined using the Pierce™ BCA Protein Assay Kit (*n* = 6; Thermo Fisher Scientific, Rockford, IL, USA) according to the manufacturer’s protocol (see Table S2). Levels of lipid peroxidation and the activities of selected antioxidant enzymes were measured in tissue homogenate supernatants using MDA Assay Kit (*n* = 3; Sigma-Aldrich, St. Louis, MO, USA), SOD Determination Kit (*n* = 5; Sigma-Aldrich, St. Louis, MO, USA), and CAT Assay Kit (*n* = 4; Sigma-Aldrich, St. Louis, MO, USA) according to the manufacturers’ instructions (see Tables S3–S5). All assays were performed using a Varioskan™ LUX multimode microplate reader.

### 4.6. Statistics

Statistical analyses were performed using GraphPad Prism version 8.3.1 (GraphPad Software, San Diego, CA, USA) and Statistica version 14 (TIBCO Software Inc., Palo Alto, CA, USA). Data were tested for normality using the Shapiro–Wilk test, and outliers were identified using Grubbs’ test (α = 0.05). Comparisons among multiple groups were performed using ANOVA followed by Dunnett’s or Tukey’s post hoc test, as appropriate. When the assumption of normality was not met, the Kruskal–Wallis test followed by Dunn’s post hoc test was applied. Comparisons between two independent groups were conducted using an unpaired *t*-test. When appropriate, two-way repeated-measures (RM) ANOVA followed by Dunnett’s multiple comparisons test was used to evaluate differences between treatment groups across multiple time points. For small sample comparisons (*n* = 5), the non-parametric Mann–Whitney test was used to confirm differences between groups, and effect size was estimated using Cohen’s d.

EC_50_ values were determined by nonlinear regression using a four-parameter logistic model (variable slope, log[agonist] vs. response) implemented in GraphPad Prism. Concentration–response curves were generated from independent experiments, and EC_50_ values are presented as mean ± standard deviation (SD).

Data are expressed as mean ± SD, and *p* < 0.05 was considered statistically significant.

## 5. Conclusions

This study demonstrates that positional O-methylation is a key determinant of flavonol bioactivity, differentially affecting antioxidant capacity, COX-2 interaction, and organism-level responses. Methylation at the C3′ position reduces radical-scavenging efficiency while possibly favoring interactions within the COX-2 catalytic channel, whereas methylation at the C7 position preserves the catechol system and is associated with stronger redox-buffering properties under the applied experimental conditions. Consequently, within the conditions of the present study, isorhamnetin exhibited stronger COX-2 inhibitory activity, while rhamnetin showed superior antioxidant performance, lower developmental toxicity, and a more balanced functional profile under oxidative stress conditions. Both flavonols attenuated 6-OHDA-associated locomotor deficits and modulated antioxidant enzyme activity in zebrafish larvae. Under the applied experimental conditions, rhamnetin induced fewer observable developmental abnormalities than isorhamnetin. Importantly, the obtained results support a structure–activity framework linking flavonol methylation pattern with redox regulation, inflammatory modulation, endogenous antioxidant defense, and behavioral outcomes. Thus, the observed neuroprotective-related effects likely arise from coordinated modulation of oxidative stress and neuroinflammatory pathways rather than from a single dominant mechanism.

Nevertheless, molecular docking remains predictive in nature, and the zebrafish larval model does not fully reproduce mammalian neuropathology. Therefore, the present study provides mechanistic insight into how subtle positional differences in O-methylation shape flavonol bioactivity across molecular, biochemical, and organismal levels, supporting the relevance of methylated flavonols as promising lead structures for phytopharmaceutical strategies targeting neurodegenerative disorders. However, regarding modulation of neuroinflammatory pathways, further studies involving direct target validation, inflammatory profiling, dopaminergic neuronal markers, and mammalian models are required to confirm translational relevance and fully assess the therapeutic risk–benefit profile of the investigated flavonols.

## Figures and Tables

**Table 1:** Half-maximal effective concentrations (EC_50_) values of isorhamnetin and rhamnetin in the DPPH, ABTS, FRAP, and CUPRAC assays.

Method	EC_50_ [µM]		*n*	*p*
	Isorhamnetin	Rhamnetin		
DPPH	31.61 ± 7.14	14.88 ± 3.00	4	0.0050 *
ABTS	15.26 ± 3.32	6.41 ± 1.22	4	0.0011 *
FRAP	141.61 ± 40.68	55.73 ± 11.61	5	0.0014 *
CUPRAC	90.56 ± 15.43	74.60 ± 27.17	5	0.2890

EC_50_ values were calculated from concentration–response curves obtained in independent experiments involving 2,2-diphenyl-1-picrylhydrazyl (DPPH) radical scavenging, 2,2′-azinobis-(3-ethylbenzothiazoline-6-sulphonic acid) (ABTS) radical cation scavenging, cupric reducing antioxidant capacity (CUPRAC), and ferric reducing antioxidant power (FRAP) assays and are presented as mean ± standard deviation (SD); * *p*-values < 0.01 were considered statistically significant.

## Data Availability

The raw data supporting the conclusions of this article will be made available by the authors on request.

## Supplementary Materials

The following supporting information can be downloaded at: https://www.mdpi.com/article/10.3390/ijms27114988/s1.

## Abbreviations

The following abbreviations are used in this manuscript:

6-OHDA	6-hydroxydopamine
ABTS	2,2′-azinobis-(3-ethylbenzothiazoline-6-sulphonic acid)
AD	Alzheimer’s disease
ANOVA	One-way analysis of variance
BCA	Bicinchoninic Acid
BHT	Butylated hydroxytoluene
CAT	Catalase
cLogP	Calculated logarithm of the octanol/water partition coefficient
COX-2	Cyclooxygenase-2
CUPRAC	Cupric reducing antioxidant capacity
DMSO	Dimethyl sulfoxide
dpf	Days post fertilization
DPPH	2,2-diphenyl-1-picrylhydrazyl
EC_50_,	Half-maximal effective concentrations
FRAP	Ferric reducing antioxidant power
HAT	Hydrogen atom transfer
IC_50_	Half-maximal inhibitory concentration
IL	Interleukins
LC50	Median lethal concentration
MDA	Malondialdehyde
NO	Nitric oxide
NSAID	Non-steroidal anti-inflammatory drug
PD	Parkinson’s disease
PG	Prostaglandins
ROS	Reactive oxygen species
SET	Single-electron transfer
SOD	Superoxide dismutase
TPSA	Topological polar surface area
TNF-α	Tumor Necrosis Factor α
TPTZ	2,4,6-tripyridyl-S-triazine
