# Supplementary material for: Structure–Activity Relationship of Flavonol O-Methylation Revealed by In Vitro, In Silico and Zebrafish Neurodegeneration Models
Source: Int J Mol Sci. 2026 May 30;27(11):4988. doi: 10.3390/ijms27114988 (PMC13257096; doi:10.3390/ijms27114988)
Supplement: Supplementary file 1 [file ijms-27-04988-s001.zip › ijms-4322315-supplementary.pdf]

Supplementary materials

Structure–Activity Relationship of Flavonol O-Methylation Revealed by *In Vitro*, *In Silico* and Zebrafish Neurodegeneration Models

Table S1. Human recombinant cyclooxygenase-2 (COX-2) inhibitor screening assay

Procedure	Calculations
10 µL standard (10 – 20 mM in EtOH)<br>+ 160 µL Tris-HCL buffer (100 mM, pH 8.0, containing 5 mM ethylenediaminetetraacetic acid (EDTA) and 2mM phenol)<br>+ 10 µL heme [in dimethylsulfoxide (DMSO), dissolved in 100 mM Tris-HCL]<br>+ 10 µL COX-2 (in 100 mM Tris-HCL)	The anti-COX activity was calculated for the highest concentration (20 mM; final concentration: 870 µM) using the calibration curve of prostaglandin standards (the working solutions: 3.9–500 pg/mL);<br>$y = -0.153\ln(x) + 1.1276$<br>$R^2 = 0.9742$
Incubation (37 °C, 30 min)	
+ 10 µL arachidonic acid (200 µM fortified with 100 mM potassium hydroxide, 1:1, in ultrapure water)	
Incubation (37 °C, 2 min)	
+ 30 µL HCl (a saturated solution; the reduction of prostaglandin H2 to prostaglandin F2α)	
Incubation (37 °C, 15 min)	
50 µL diluted standards samples (1:2000 and 1:4000) were incubated with 50 µL tracer and 50 µL antiserum for 18 hours a 4 °C.<br>Development of the plate: The plate was washed (5 times with buffer) and 200 µL 5,5′-dithiobis(2-nitrobenzoic acid (DTNB) was added.	
After 90 minutes of shaking the reaction mixtures with DTNB in the covered plate, absorbance was read (412 nm, Varioskan™ LUX multimode microplate reader).	

Table S2. Pierce™ bicinchoninic acid (BCA) protein assay kit

BCA assay	25 µL sample<br>+ 200 µL Working Reagent (the mix of 20 mL BCA Reagent A and 400 µL BCA Reagent B)	The protein concentration was calculated using the calibration curve of bovine serum albumin (BSA).<br>A volume of 25 µL of working solutions (0–1000 µg/mL) were added to the reaction mixture to obtain final concentrations ranging from 0 to 100 µg/mL;<br>$y = 0.0132x - 0.0001$<br>$R^2 = 1$
	The absorbance at 562 nm was read after 30 min of incubation on a shaker at 37 °C (Varioskan™ LUX multimode microplate reader)	

Table S3. Lipid peroxidation with malondialdehyde (MDA) assay kit

MDA assay	100 µL sample<br>+ 300 µL thiobarbituric acid (TBA)	The MDA concentrations were calculated using the calibration curve.<br>A volume of 100 µL of working solutions (0–8 nM) were added to the reaction mixture to obtain final concentrations ranging from 0 to 2 nM;<br>$y = 1.2503x - 0.0119$<br>$R^2 = 0.9996$
	The tubes we incubated on a shaker for 60 min at 95 °C, followed by cooling in an ice bath for 10 min. Subsequently, every 180 µL of the each reaction mixture were transferred to a 96-well microplate, and the absorbance at 532 nm was read (Varioskan™ LUX multimode microplate reader)	

Table S4. Superoxide dismutase (SOD) determination assay kit

SOD activity	10 μL sample<br>+ 200 μL of 2-(4-iodophenyl)-3-(4-nitrophenyl)-5-(2,4-disulfophenyl)-2H-tetrazolium, monosodium salt (WST) working solution (1 mL of WST solution was reconstituted with 19 mL of buffer solution)<br>+ 20 μL enzyme working solution (15 μL of Enzyme Solution was diluted with 2.5 mL of Dillution Buffer)	SOD activity was calculated using the calibration curve of SOD (catalog number S7571).<br>A volume of 10 μL of working solutions (0–1 U/mL) were added to the reaction mixture to obtain final concentrations ranging from 0.000 to 0.043 U/mL;<br>$y = -5.6769x + 0.5683$<br>$R^2 = 0.9834$
	After incubation (37 °C, 20 min), absorbance was read (450 nm, Varioskan™ LUX multimode microplate reader)	

Table S5. Catalase (CAT) activity assay kit

CAT activity	20 μL sample<br>+ 100 μL potassium phosphate buffer (100 mM, pH 7.0)<br>+ 30 μL methanol<br>+ 20 μL H_2O_2 (35.28 mM, in ultrapure water)	The CAT activity was calculated using the calibration curves of formaldehyde.<br>A volume of 20 μL of working solutions (0–60 μM) were added to the reaction mixture to obtain final concentrations ranging from 0 to 5.71 μM;<br>$y = 0.1151x + 0.0152$<br>$R^2 = 0.981$
	A covered plate was incubated on a shaker for 20 min at room temperature. To terminate the reaction, 30 μL of 10 M potassium hydroxide (in ultrapure water) was added.	
	+ 30 μL Purpald (in 0.5 M HCl)	
	The covered plate was once again incubated on a shaker for 10 min at room temperature.	
	+ 10 μL potassium periodate in 0.5 M potassium hydroxide	
	The absorbance at 540 nm was read after 5 min of shaking (Varioskan™ LUX multimode microplate reader)	

A

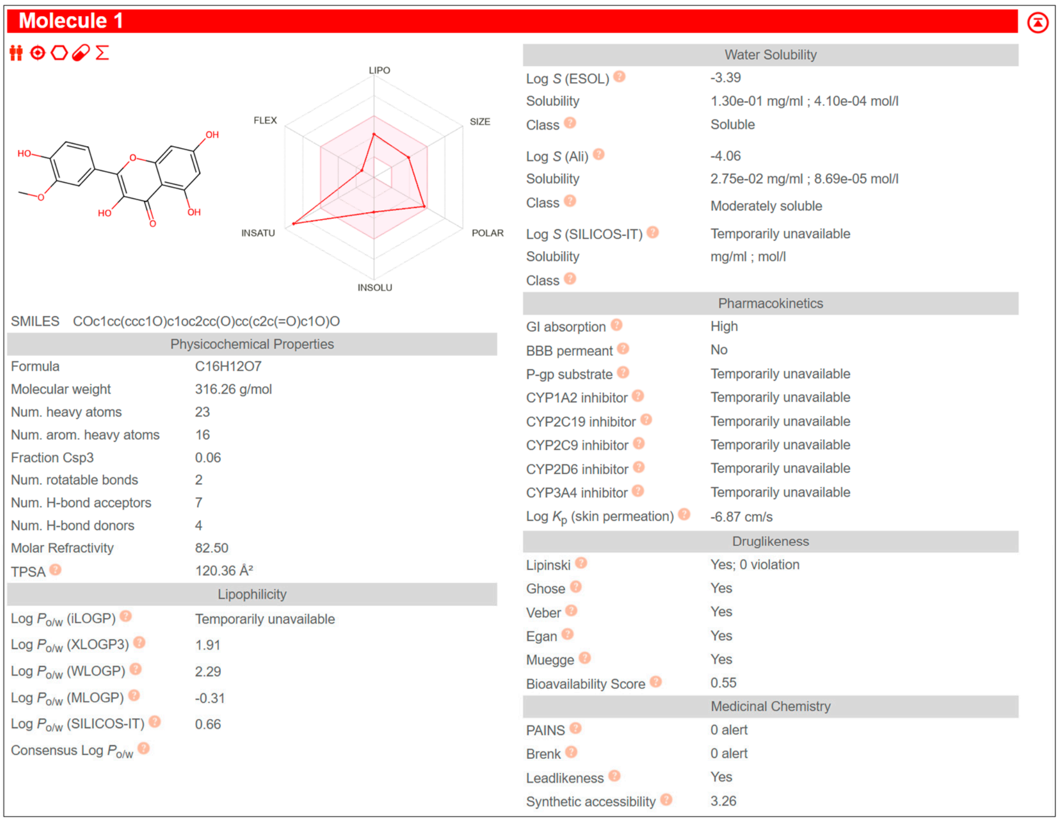

SMILES COc1cc(ccc1O)c1oc2cc(O)cc(c2c(=O)c1O)O

Physicochemical Properties	
Formula	C16H12O7
Molecular weight	316.26 g/mol
Num. heavy atoms	23
Num. arom. heavy atoms	16
Fraction Csp3	0.06
Num. rotatable bonds	2
Num. H-bond acceptors	7
Num. H-bond donors	4
Molar Refractivity	82.50
TPSA	120.36 Å²

Lipophilicity	
Log $P_{o/w}$ (iLOGP)	Temporarily unavailable
Log $P_{o/w}$ (XLOGP3)	1.91
Log $P_{o/w}$ (WLOGP)	2.29
Log $P_{o/w}$ (MLOGP)	-0.31
Log $P_{o/w}$ (SILICOS-IT)	0.66
Consensus Log $P_{o/w}$	

Water Solubility	
Log *S* (ESOL)	-3.39
Solubility	1.30e-01 mg/ml ; 4.10e-04 mol/l
Class	Soluble
Log *S* (Ali)	-4.06
Solubility	2.75e-02 mg/ml ; 8.69e-05 mol/l
Class	Moderately soluble
Log *S* (SILICOS-IT)	Temporarily unavailable
Solubility	mg/ml ; mol/l
Class	

Pharmacokinetics	
GI absorption	High
BBB permeant	No
P-gp substrate	Temporarily unavailable
CYP1A2 inhibitor	Temporarily unavailable
CYP2C19 inhibitor	Temporarily unavailable
CYP2C9 inhibitor	Temporarily unavailable
CYP2D6 inhibitor	Temporarily unavailable
CYP3A4 inhibitor	Temporarily unavailable
Log K_p (skin permeation)	-6.87 cm/s

Druglikeness	
Lipinski	Yes; 0 violation
Ghose	Yes
Veber	Yes
Egan	Yes
Muegge	Yes
Bioavailability Score	0.55

Medicinal Chemistry	
PAINS	0 alert
Brenk	0 alert
Leadlikeness	Yes
Synthetic accessibility	3.26

B

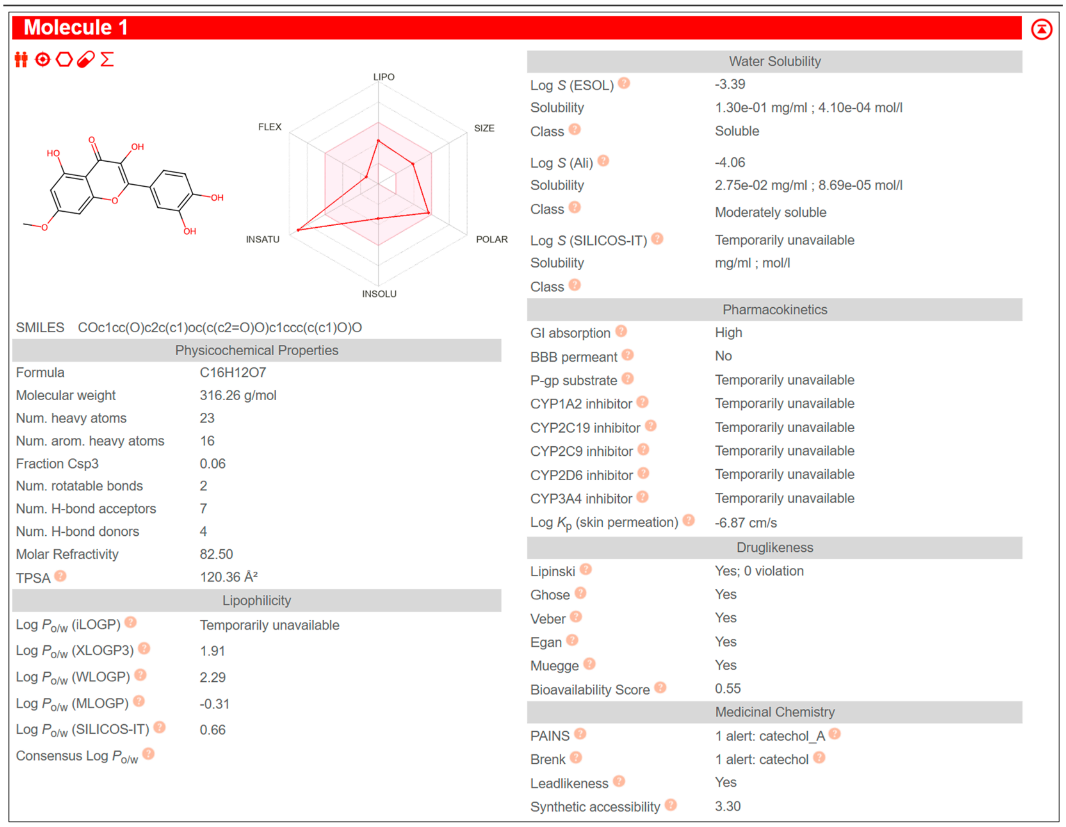

SMILES COc1cc(O)c2c(c1)oc(c(c2=O)O)c1ccc(c(c1)O)O

Physicochemical Properties	
Formula	C16H12O7
Molecular weight	316.26 g/mol
Num. heavy atoms	23
Num. arom. heavy atoms	16
Fraction Csp3	0.06
Num. rotatable bonds	2
Num. H-bond acceptors	7
Num. H-bond donors	4
Molar Refractivity	82.50
TPSA	120.36 Å²

Lipophilicity	
Log $P_{o/w}$ (iLOGP)	Temporarily unavailable
Log $P_{o/w}$ (XLOGP3)	1.91
Log $P_{o/w}$ (WLOGP)	2.29
Log $P_{o/w}$ (MLOGP)	-0.31
Log $P_{o/w}$ (SILICOS-IT)	0.66
Consensus Log $P_{o/w}$	

Water Solubility	
Log *S* (ESOL)	-3.39
Solubility	1.30e-01 mg/ml ; 4.10e-04 mol/l
Class	Soluble
Log *S* (Ali)	-4.06
Solubility	2.75e-02 mg/ml ; 8.69e-05 mol/l
Class	Moderately soluble
Log *S* (SILICOS-IT)	Temporarily unavailable
Solubility	mg/ml ; mol/l
Class	

Pharmacokinetics	
GI absorption	High
BBB permeant	No
P-gp substrate	Temporarily unavailable
CYP1A2 inhibitor	Temporarily unavailable
CYP2C19 inhibitor	Temporarily unavailable
CYP2C9 inhibitor	Temporarily unavailable
CYP2D6 inhibitor	Temporarily unavailable
CYP3A4 inhibitor	Temporarily unavailable
Log K_p (skin permeation)	-6.87 cm/s

Druglikeness	
Lipinski	Yes; 0 violation
Ghose	Yes
Veber	Yes
Egan	Yes
Muegge	Yes
Bioavailability Score	0.55

Medicinal Chemistry	
PAINS	1 alert: catechol_A
Brenk	1 alert: catechol
Leadlikeness	Yes
Synthetic accessibility	3.30

C

Molecule 1

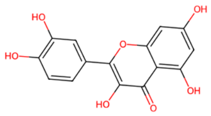

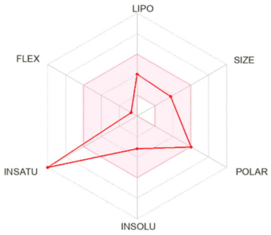

SMILES Oc1cc(O)c2c(c1)oc(c(c2=O)O)c1ccc(c(c1)O)O

Physicochemical Properties	
Formula	C15H10O7
Molecular weight	302.24 g/mol
Num. heavy atoms	22
Num. arom. heavy atoms	16
Fraction Csp3	0.00
Num. rotatable bonds	1
Num. H-bond acceptors	7
Num. H-bond donors	5
Molar Refractivity	78.03
TPSA	131.36 Å²

Lipophilicity	
Log $P_{o/w}$ (iLOGP)	Temporarily unavailable
Log $P_{o/w}$ (XLOGP3)	1.59
Log $P_{o/w}$ (WLOGP)	1.99
Log $P_{o/w}$ (MLOGP)	-0.56
Log $P_{o/w}$ (SILICOS-IT)	0.14
Consensus Log $P_{o/w}$	

Water Solubility	
Log *S* (ESOL)	-3.19
Solubility	1.96e-01 mg/ml ; 6.49e-04 mol/l
Class	Soluble
Log *S* (Ali)	-3.96
Solubility	3.32e-02 mg/ml ; 1.10e-04 mol/l
Class	Soluble
Log *S* (SILICOS-IT)	Temporarily unavailable
Solubility	mg/ml ; mol/l
Class	

Pharmacokinetics	
GI absorption	High
BBB permeant	No
P-gp substrate	Temporarily unavailable
CYP1A2 inhibitor	Temporarily unavailable
CYP2C19 inhibitor	Temporarily unavailable
CYP2C9 inhibitor	Temporarily unavailable
CYP2D6 inhibitor	Temporarily unavailable
CYP3A4 inhibitor	Temporarily unavailable
Log K_p (skin permeation)	-7.01 cm/s

Druglikeness	
Lipinski	Yes; 0 violation
Ghose	Yes
Veber	Yes
Egan	Yes
Muegge	Yes
Bioavailability Score	0.55

Medicinal Chemistry	
PAINS	1 alert: catechol_A
Brenk	1 alert: catechol
Leadlikeness	Yes
Synthetic accessibility	3.23

Figure S1. SwissADME results for (A) Isorhamnetin; (B) Rhamnetin; (C) Quercetin (accessed at: https://www.swissadme.ch/index.php, on 21 May 2026)
